# Challenges of current treatment and exploring the future prospects of nanoformulations for treatment of atopic dermatitis

**DOI:** 10.1007/s43440-023-00510-3

**Published:** 2023-09-05

**Authors:** Vandita Kakkar, Komal Saini, Kamalinder K. Singh

**Affiliations:** 1 Department of Pharmaceutics, University Institute of Pharmaceutical Sciences, Panjab University, Chandigarh, 160014 India; 2 School of Pharmacy and Biomedical Sciences, Faculty of Clinical and Biomedical Sciences, University of Central Lancashire, Preston, PR1 2HE Lancashire UK; 3 UCLan Research Centre for Smart Materials, University of Central Lancashire, Preston, PR1 2HE Lancashire UK; 4 UCLan Research Centre for Translational Biosciences and Behaviour, University of Central Lancashire, Preston, PR1 2HE Lancashire UK

**Keywords:** Atopic dermatitis, Topical delivery, Nanoformulation, Nanoparticles, Skin penetration, Safety aspects

## Abstract

Atopic dermatitis (AD) is a predominant and deteriorating chronic inflammation of the skin, categorized by a burning sensation and eczematous lesions in diverse portions of the body. The treatment of AD is exclusively focused to limit the itching, reduce inflammation, and repair the breached barrier of the skin. Several therapeutic agents for the treatment and management of AD have been reported and are in use in clinics. However, the topical treatment of AD has been an unswerving challenge for the medical fraternity owing to the impaired skin barrier function in this chronic skin condition. To surmount the problems of conventional drug delivery systems, numerous nanotechnology-based formulations are emerging as alternative new modalities for AD. Latter enhances the bioavailability and delivery to the target disease site, improves drug permeation and therapeutic efficacy with reduced systemic and off-target side effects, and thus improves patient health and promotes compliance. This review aims to describe the various pathophysiological events involved in the occurrence of AD, current challenges in treatment, evidence of molecular markers of AD and its management, combinatorial treatment options, and the intervention of nanotechnology-based formulations for AD therapeutics.

**Graphical abstract:**

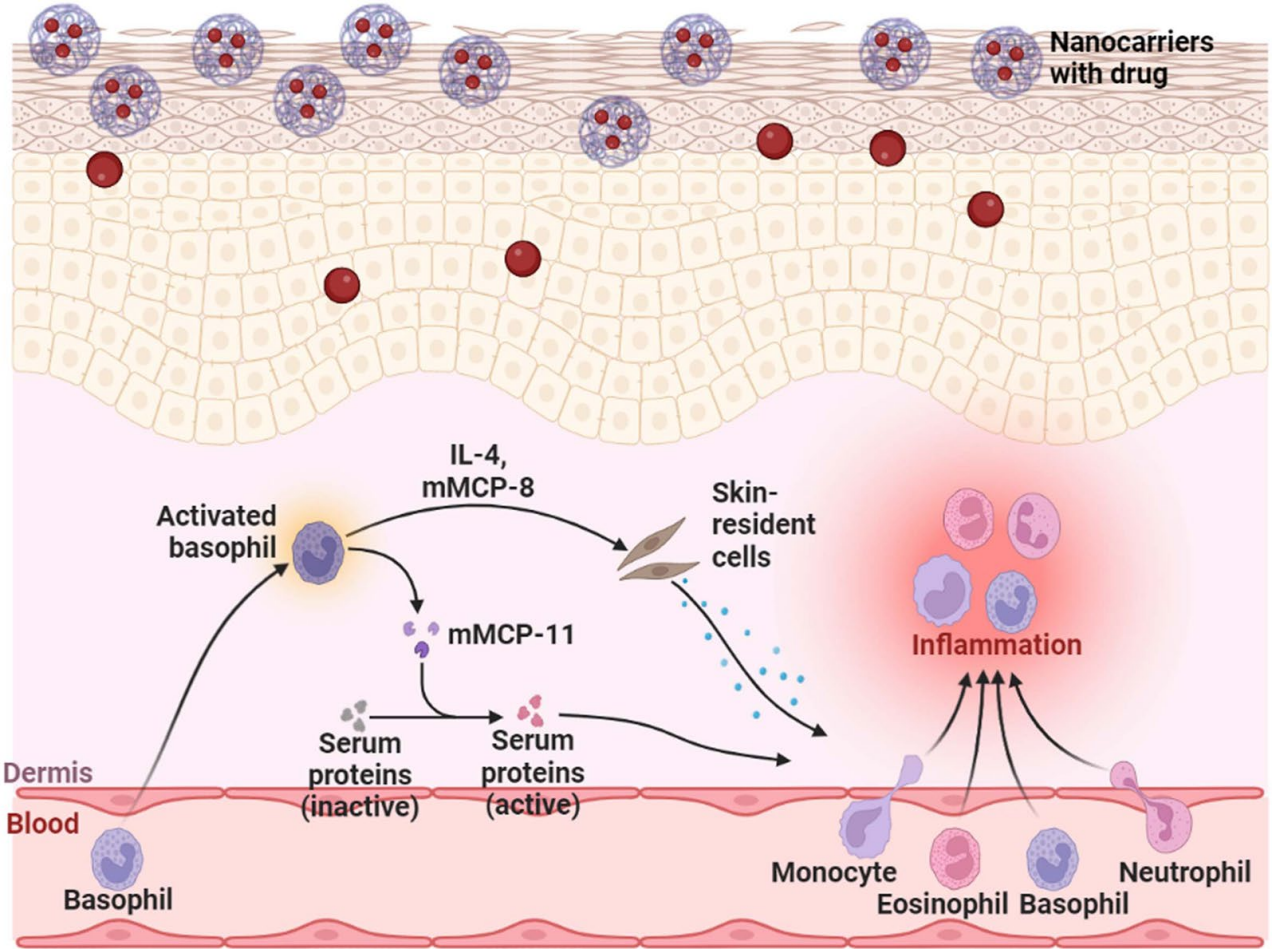

## Introduction

Atopic dermatitis (AD) is a common chronic inflammatory skin disease characterized by eczematous lesions, itching, redness, and flaking of the skin [1]. It is a prevalent long-term condition that affects a significant portion of the global population. Recent studies have shown a continuous increase in the incidence of AD, particularly among urban children (15–30%) and adults (1–3%) [2–4]. This significant rise in prevalence over the past three decades has raised concerns, and there is evidence suggesting that environmental factors contribute to the development of AD [5]. AD is not limited to a specific age group but can affect individuals of any age. It often begins in early childhood and may recur throughout a patient’s life [6]. There is a familial predisposition to AD, with a higher prevalence of atopic symptoms such as allergic rhinitis, bronchial asthma, and food allergies among affected individuals [7].

**Fig. 1:**
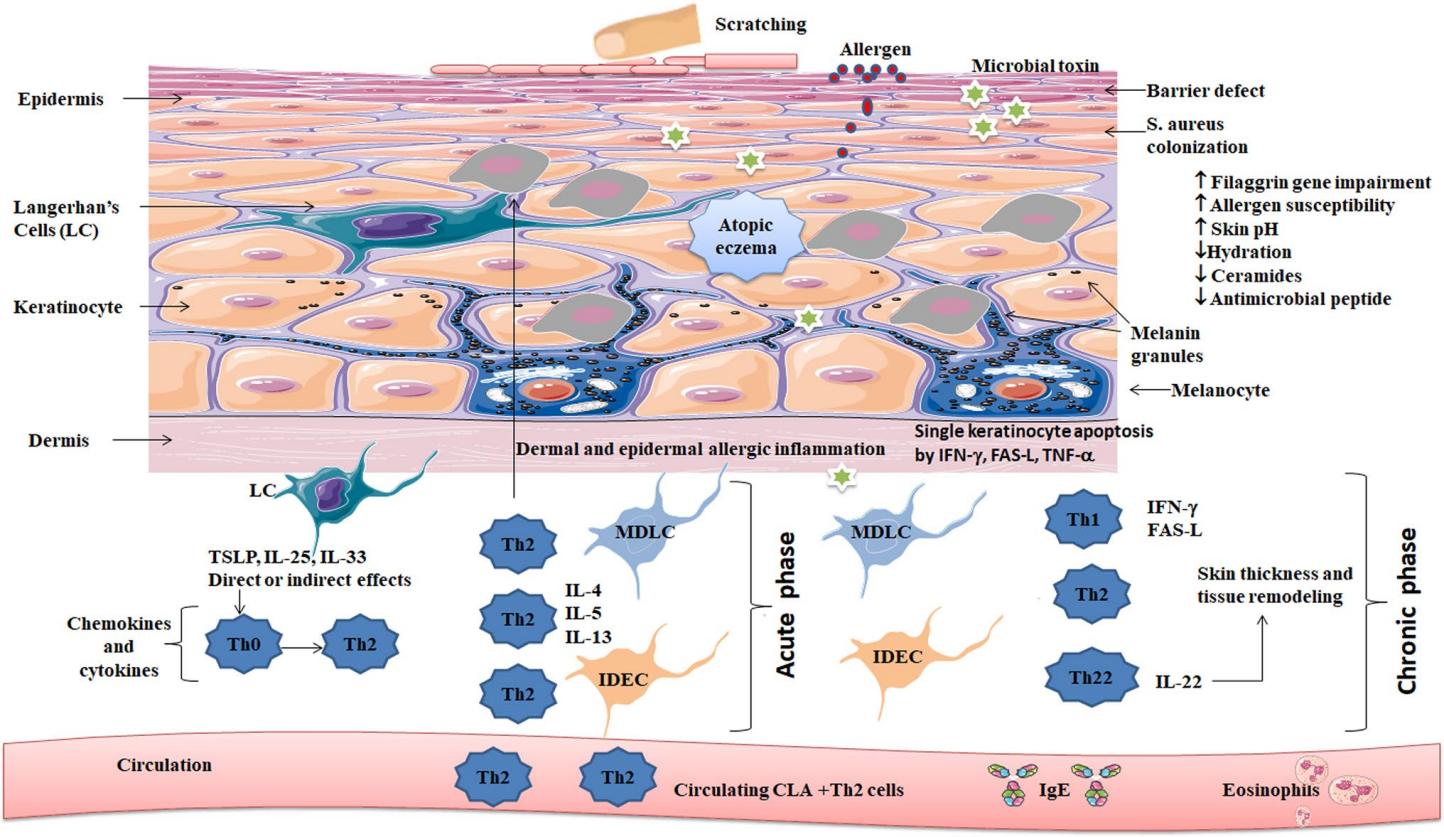
Current understanding of the pathogenic processes in acute atopic dermatitis (AD) and chronic AD. Antigen-presenting cells including Langerhans cells (LCs) monocyte-derived LC-like cells (MDLC), and inflammatory dendritic epidermal cells (IDECs) exist in the skin. IDECs and LC-like cells have been found to be present in both steady states and inflammatory states, and also present in lesional AD. The key cytokines found in AD, interleukin (IL)-4, 5, 13, and 22 play a similar role in acute and chronic stages of the disease. Interferon (IFN), immunoglobulin-E (IgE), IL, T helper (Th) lymphocytes, and thymic stromal lymphopoietin (TSLP) are found in the diagram with their expression

AD is a multifactorial disorder characterized by non-contagious exudative eczema, primarily caused by a disruption of the stratum corneum barrier (SC). This disruption leads to impaired skin function and increased transepidermal water loss (TEWL), resulting in dehydration. In addition, AD involves various inflammatory processes characterized by the release of cytokines, chemokines, and interleukins (IL) such as IL-1, IL-4, IL-5, IL-6, IL-8, IL-10, IL-12p70, and IL-13. These molecular markers, along with tumor necrosis factor-alpha (TNF-α) and interferon-gamma (IFN-γ), contribute to lipogenesis, generating active lipids crucial for both skin barrier function and the distribution of biochemical signals. Consequently, these factors exacerbate inflammation and increase the risk of infections (Fig. 1). AD is also associated with dysregulation in apoptotic cascades.

Furthermore, high concentrations of elastase have been detected in peripheral blood neutrophils in individuals with AD. This disparity between proteolytic enzyme levels and their endogenous inhibitors leads to the disruption of elastic fiber organization [9, 10]. Topical application of drugs remains as a common route for AD treatment. Nonetheless, a variety of pharmacological and non-pharmacological approaches, such as detecting and avoiding causative allergens, skin hydration (e.g., baths or moisturizers), topical anti-inflammatory or immunosuppressive treatments (tacrolimus and pimecrolimus), anti-pruritic drugs (corticosteroids), and anti-bacterial agents (e.g., bleach baths, applying antiseptics or disinfectants) have shown to be effective for AD treatment with mild to severe symptoms, either alone or in combination with other modalities.

Topical formulations including the corticosteroids and calcineurin inhibitors for AD and other inflammatory conditions act by inhibiting the activation of calcineurin in T-cells, and decreasing the secretion of pro-inflammatory cytokines and mediators involved in the AD process. However, they are associated with skin burning, warmth, redness, and local allergic reactions. Further, there is also a risk of secondary infections due to reduced immunity. In addition, systemic absorption of these medications can lead to breathing difficulties, facial swelling, and systemic immunosuppressive effects, increasing the risk of infections and malignancies. Moreover, the absorption rates and therapeutic efficiencies of ointments like tacrolimus and pimecrolimus is also reported to be variable. To address these concerns, nanotechnology can be employed to target inflamed skin areas specifically, thereby avoiding risks to healthy skin and systemic circulation.

Newer approaches for AD treatment are, therefore, urgently needed which could direct towards improving the structural skin abnormalities and immune dysregulation which are significant pathogenic events. As a result, optimal AD management needs a multifaceted strategy aimed at healing the diseased site and providing a shield across the skin barrier, and addressing the complex immunopathogenesis [11]. There is still no promising therapeutic regimen that might overturn the pathological effects of AD. However, topical nanotechnology-based formulations seem to possess a presumed ability to surmount the above-mentioned limitations.

Nanotechnology, a rapidly expanding field with applications in health and disease, is being explored to develop safe and effective therapies to reduce the symptoms of AD post topical and systemic administration. Novel nanoparticle-based systems have shown promise for dermal delivery due to their ability to enhance drug permeation across the SC, which is naturally not permeable to a range of substances, as skin acts as a natural physical barrier to particle penetration. Topical nanocarrier-based formulations have the potential to enhance skin targeting and increase drug efficacy and decrease systemic side effects [2–4]. Even though nanoparticle drug delivery has been hailed as a game-changer, its potential for treating localized skin and systemic disorders has yet to be realized [12]. This article will present an exploration of pathophysiology, current solutions, safety aspects, and futuristic viewpoints of nanoformulations for AD.

## Barrier function in the pathogenesis of atopic dermatitis

The skin barrier plays a vital role in protecting the body from external threats such as pathogens, chemicals, irritants, and allergens. It acts as a defense mechanism, preventing these substances from penetrating deeper layers of the skin, including the epidermis and dermis, where they could potentially trigger an immune response. In addition, the skin barrier serves to retain moisture and prevent excessive water loss through the epidermal layer. The function of the skin barrier can be assessed by measuring the percentage of transepidermal water loss (%TEWL), which is inversely related to the diffusional permeation path length through the stratum corneum. Various methods can be employed to evaluate skin barrier function. These include measuring the pH of the skin surface, assessing the permeation of tracer compounds, and evaluating the cohesion and hydration of the stratum corneum [13]. These techniques provide valuable insights into the integrity and effectiveness of the skin barrier.

### The stratum corneum (SC)

The uppermost layer, known as the ‘brick and mortar’ of the skin of the epidermis is the SC. It comprises of highly organized intercellular lipid matrix which acts as mortar and corneocytes referred to as brick (keratinocytes without nuclei and cytoplasmic organelles) [13]. There are altered SC homeostasis in patients having AD with lesional and non-lesional skin. This causes an enhancement in water loss and increased access to allergens [14].

The structure and composition of the SC in AD condition can be influenced in the subsequent ways [14]:

- Loss or decreased function of filaggrin protein (FLG)
- Enhanced serine protease activity
- Damaged lipid processing.

### Filaggrin (FLG) protein

FLG protein plays an important role in the structural integrity of the SC. FLG accumulates the keratin filaments within the corneocytes and then facilitates to the formation of a cornified cell envelope adjacent to corneocytes. FLG additionally provides SC with a water-holding capacity and acidic pH. The maintenance of acidic pH is essential to control the activity of the enzyme that shows desquamation, production of lipids, and inflammation [13, 15]. A loss-of-function mutation in the FLG gene represents a significant hereditary risk factor in AD. FLG mutations are linked to a history of AD onset, disease severity, and disease persistence. About 50% of AD cases with moderate-to-severe symptoms can be ascribed to FLG mutations [15].

### Serine protease activity and pH

Kallikrein (KLK) 5 and 7 are the serine proteases, and their activity is adjusted by the pH of the stratum corneum. Both proteases KLK5 and KLK7 break the extracellular corneodesmosomal proteins that simultaneously connect the corneocytes. Their elevated activity shows decreased corneocyte adhesion and desquamation [16]. The pH of skin is normally acidic and limits the protease activity (more active in basic medium). In AD, the skin pH is increased and consequently reflects in the increased activity of serine protease [17].

Another enzyme that is involved in AD disease is the neutrophil elastase (NE), which is a broad-spectrum serine protease released from neutrophils and macrophages. It can degrade several proteins of the extracellular matrix, acting as a potent pro-inflammatory agent and generating chemotactic factors. Following the matrix degradation and loss of intercellular contacts, NE appears to participate in the inflammatory features of eczematous diseases resulting in spongiosis and desquamation. Particularly, NE activity has been shown to be absent in the skin of healthy individuals, whereas a massive increase in its action has been demonstrated in the skin of patients with allergic contact (55-fold average) and atopic dermatitis (35-fold), the most common form of eczema [17].

### Lipid matrix function

Cholesterol, free fatty acids, and ceramides are the three main types of lipids present in SC. This lipid matrix forms a highly ordered structure of compactly packed layers of lipid. This is the most important passageway for substances traversing the intercellular lipid matrix across the skin barrier [13]. In AD, a decrease in total lipids and a change in their composition have been observed. As a result of this impaired skin barrier function, enhanced penetrability of the drugs via stratum corneum has been observed [13].

### The immune system and skin barrier function

Skin barrier damage can assist the transportation of allergens or haptens (the molecules that only bring out an allergic effect after protein binding) into the skin structure resulting in its inflammation after stimulating the pro-inflammatory cytokines production [13]. Simultaneously, the Th2 cytokines, i.e., IL-4, IL-13, and IL-22 downregulate the FLG expression, causing further impairment to the skin barrier [18, 19].

### Skin microbiome and skin barrier function

The pathogenic and commensal bacteria, fungi, and viruses are the cutaneous microbiome that are inhabitants of our skin and help to sustain epidermal homeostasis [14]. When the stratum corneum layer is structurally proficient with a well-ordered lipid matrix, it stops the colonization of pathogens for instance *Staphylococcus aureus* (*S. aureus)*, that can impair the barrier function adversely. In AD, skin is colonized with (*S. aureus*) in more than 90% of patients [14]. *S. aureus* surface proteins decrease the production of free fatty acid in the epidermis that promotes permeability through the skin. In AD patients, *S. aureus* exotoxins with super-antigenic activities stimulate the production of IgE and produce the symptoms of pruritus. Later excoriations (skin-picking) due to itch generate further damage to the skin barrier functions [16].

#### Permeation pathways across the skin

**Fig. 2:**
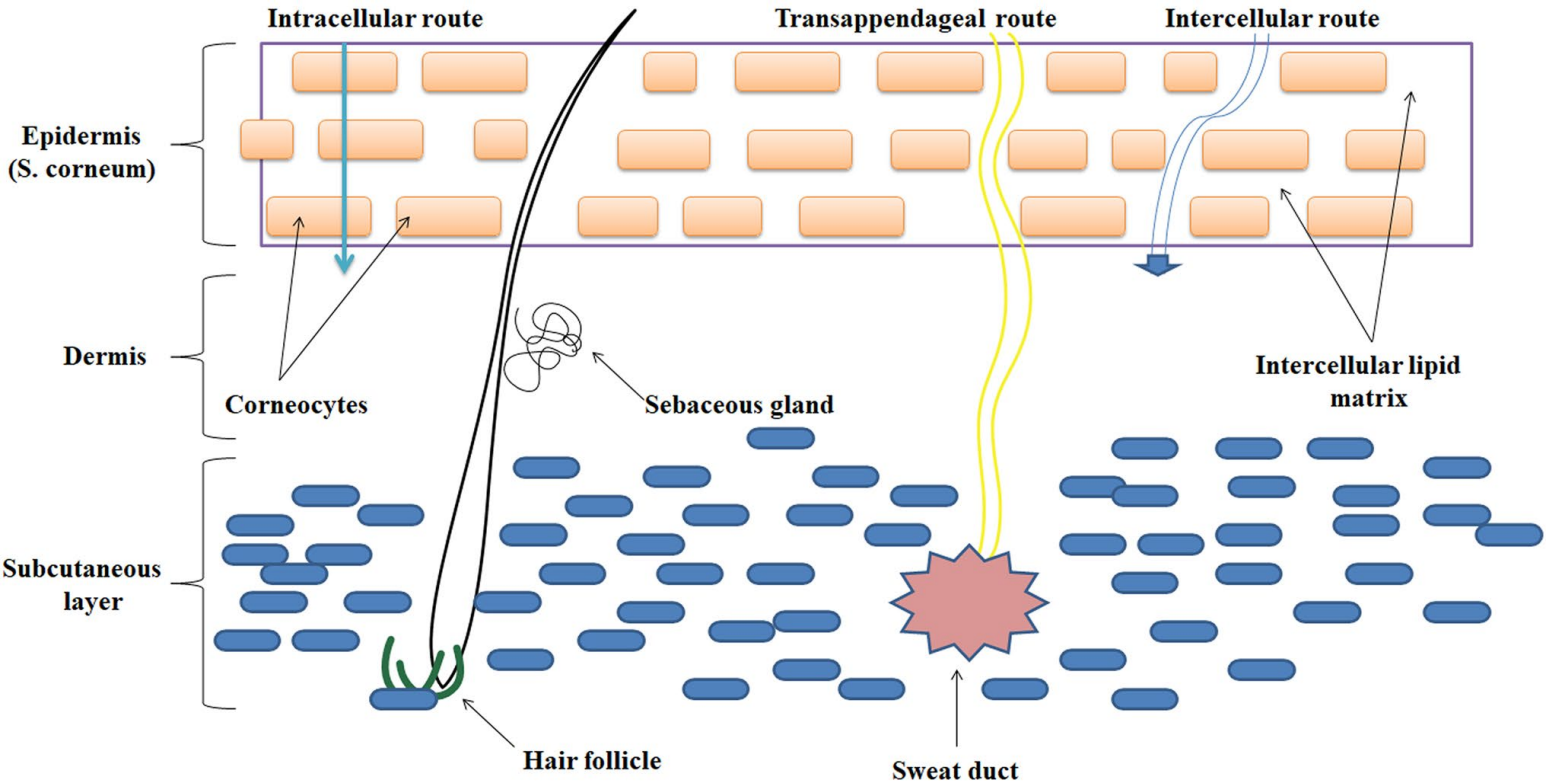
Permeation pathways across the skin

Molecules can traverse the skin through two main pathways: the transepidermal pathway, which involves diffusion across the layers of the skin, and the transappendageal route, which utilizes hair follicles or sweat ducts. The overall movement of molecules across the skin is determined by the combined flux of these two pathways, as illustrated in Fig. 2.

### Transepidermal pathway

The movement of a permeant across the skin occurs through both intracellular and extracellular spaces along the transepidermal pathway, starting from the epidermis and progressing to the dermis and then the hypodermis. The permeant can move within cells as well as between cells. To traverse the alternating layers of cells and extracellular matrix, the permeant undergoes a combination of cellular divisions and diffusions, which can be categorized into hydrophilic and lipophilic zones. There are variations in the hydrophilic and lipophilic properties of cells and substances between different skin layers. However, in general, the interiors of cells tend to be more hydrophilic compared to the extracellular matrix. As the permeant moves through the skin layers, it navigates the convoluted intercellular channels within the extracellular matrix without passing through individual cells. Lipophilic molecules prefer the intercellular pathway, while small hydrophilic molecules tend to favor the transcellular route [20].

### Transappendageal pathway

The permeation of substances through hair follicles, known as the transfollicular route, or sweat ducts is considered part of the appendageal or shunt route. However, the exact mechanism and extent of transfollicular permeation for topically applied agents have not been fully elucidated to date. Transfollicular penetration typically occurs in the area of the infundibulum, particularly in the lower section where the barrier function may be compromised due to changes in the differentiation pattern. In intact skin, intercellular or transfollicular permeation has not been observed for particles larger than 100 nm. However, further research is necessary to determine a clear threshold for smaller particles below which transfollicular and intercellular penetration can occur. This aspect is crucial for risk assessment and requires additional investigation [21].

### Relative involvements of permeation pathways

The primary pathway for compounds to penetrate the skin is the transepidermal pathway, with the movement of substances across the SC layer serving as the rate-limiting step, influencing the overall flow of the permeant under sink conditions. Previous research has shown that the flux of permeants through the skin does not correlate with the density of appendages on the skin surface. While the transappendageal pathway, which utilizes appendages such as hair follicles, sweat ducts, and sebaceous glands, constitutes only about 0.1% of the skin surface area (though this percentage may be higher in specific regions such as the forehead) [22], its contribution to percutaneous transport is generally considered secondary.

However, the relative importance of these pathways can vary depending on the physicochemical properties of the permeant and the specific formulation being studied. Lipophilic drugs have a tendency to accumulate in the SC, which can hinder their partitioning into the more hydrophilic viable epidermis. As a result, the clearance from the SC, rather than diffusion across it, may become the limiting factor for highly lipophilic medications. On the other hand, for highly hydrophilic compounds like caffeine and electrolytes, as well as large molecules with low diffusion coefficients, the transappendageal pathway may play a more significant role. Such compounds are effectively excluded from the transepidermal route and rely on the pathway through appendageal structures for penetration [23]. Numerous studies have demonstrated that the transappendageal pathway transiently dominates at the initial stages, but eventually gives way to the predominance of the transepidermal pathway at a steady state [22–24].

## Current approaches and challenges during the treatment of AD

### Topical pharmacotherapies

#### Corticosteroids

Topical corticosteroids (TCs) are considered the first-line treatment for AD in both adults and children. They effectively address the inflammatory signs, symptoms, acute flares, and itching associated with the disease. The efficacy of TCs in reducing both acute and chronic symptoms of AD has been well established, supported by more than 100 randomized controlled trials [25]. When selecting TCs for infants and young children, it is important to choose formulations with low systemic bioavailability and a well-established therapeutic index [25]. The choice of TC potency considers factors such as patient age, disease severity, and skin thickness/relative absorption area.

However, it is important to be aware of potential side effects associated with TCs. These side effects can include skin atrophy, perioral dermatitis, adrenal suppression, acne rosacea, and the progression of striae. When the AD lesions show improvement, it is recommended that patients gradually reduce the frequency of TC application, transitioning to maintenance therapy with a lower frequency of use. For individuals, both adults and children, with moderate-to-severe AD, long-term use of medium-potency TCs in combination with proactive twice-weekly treatment using emollients can help minimize the risk of relapse [26]. It is crucial to exercise caution and avoid regular use of high-potency TCs (such as those containing > 3% hydrocortisone) on areas of thin skin, such as the face, body folds, or groin, to mitigate the potential risk of cutaneous atrophy. For a 2-week treatment, approximately 0.5 g of the currently available cream or ointment can be applied, which corresponds to the size of two adult hands, using adult fingertip units (AFU) as a reference [27].

### Topical calcineurin inhibitors (TCIs)

Non-steroidal topical calcineurin inhibitors (TCIs), including tacrolimus and pimecrolimus, have been found to be beneficial in the treatment of acute flares and maintenance therapy for AD in both adults and children over the age of two years. TCIs exert their anti-inflammatory effects by suppressing calcineurin-dependent T-cell activation, thereby preventing the release of pro-inflammatory cytokines and mediators. While tacrolimus 0.1% ointment is indicated only for adults, tacrolimus 0.03% ointment and pimecrolimus 1% cream are recommended for patients aged two years and older with AD. However, the latest guidelines from the American Academy of Dermatology (AAD) suggest off-label use of these TCIs in children under two years with mild or severe AD [28]. The most common side effects of TCIs include temporary local burning or itching at the application site. Unlike topical steroids, TCIs do not cause skin atrophy with long-term use and help to preserve the weakened epidermal barrier [29].

Clinical trials have demonstrated the efficacy and safety of TCIs in the treatment of AD. Tacrolimus 0.1% ointment showed effectiveness over a 1-year period in children with AD, while another open-label trial revealed efficacy in adult patients with AD. In addition, a controlled clinical study conducted over six months demonstrated the effectiveness of pimecrolimus 1% cream in infants and adults with AD [30–32]. Rarely, some patients may develop allergies to these agents, and cost may be a barrier for individuals who have limited access to these topical medications. The United States Food and Drug Administration (USFDA) has issued a black box warning for TCIs, citing a theoretical risk of lymphoma based on studies in mice given high doses of the drug. However, the use of TCIs in humans does not appear to increase the incidence of lymphoma [30, 33].

TCIs are primarily recommended for the targeted treatment of AD in specific areas such as the eyelids, face, and intertriginous regions. They offer a valuable alternative for patients who experience multiple flares or have chronic AD and would otherwise require high doses of topical steroids. In cases where patients have moderate-to-severe symptoms, tacrolimus ointment (0.1%) is often used in combination with topical steroids. For individuals with mild to moderate symptoms, pimecrolimus cream (1%) is typically recommended.

While concerns have been raised about the potential risk of malignancies associated with long-term TCI use, current evidence does not indicate an increased risk of lymphoma in AD patients who have used TCIs for short to medium-term periods (over 10 years) when compared to the general population. Several studies have demonstrated that AD patients receiving maintenance therapy with tacrolimus three times a week experience fewer flares and longer intervals between relapses, providing further support for the efficacy of TCIs in managing AD [34–36].

### Wet-wrap and bleach therapy

A combined approach for treating AD flare-ups is wet-wrap therapy with topical drugs [37]. Topical agents are frequently applied to the skin, followed by a viscose tubular wet bandage layer and a secondary dry bandage layer [38]. By providing a smooth skin texture and preventing water loss, this treatment enhances the moisturizing effects [38]. Huang et al. discovered that a bleach bath combined with twice-weekly intranasal mupirocin treatment was more effective than a placebo in a trial [39]. Bleach baths have been recommended as a technique to reduce the colonization of *S. aureus* on the skin and hence prevent recurrent skin infections [40]. Antiseptics including triclosan, potassium permanganate, and chlorhexidine gluconate, as well as bleach or sodium hypochlorite, are used to treat infected skin and prevent AD. Antiseptic bathing has been demonstrated to lower the bacterial burden on the skin for patients with AD. As a result, antibiotics mixed with antiseptics have been shown to be particularly effective in treating clinically infected skin in AD [39].

During the last 2 decades, wet-wrap treatment has been advocated as a relatively safe and effective treatment modality in children with severe and/or refractory AD. Despite several publications from different research groups, there are still many unsolved issues concerning the use of wet-wrap dressings in the treatment of AD. Wet-wrap treatment using cream or ointment and a double layer of cotton bandages, with a moist first layer and a dry second layer, is an efficacious short-term intervention treatment in children with severe and/or refractory AD.

### Systemic therapy

#### Phototherapy

AAD recommends phototherapy as a topical therapy option for AD. Among the available phototherapy treatments, narrow-band ultraviolet B (UVB) is particularly advantageous due to its low-risk profile, high effectiveness, accessibility, and comfort. The Joint Task Force (JTF) guidelines further suggest using ultraviolet A (UVA) for acute exacerbations, UVB modalities for chronic AD cases, and photochemotherapy involving psoralen and UVA for AD patients with severe and widespread symptoms. Phototherapy can also serve as a maintenance therapy for individuals in the chronic phase of AD. The dosing and frequency of phototherapy depend on factors such as the minimal erythema dose, Fitzpatrick skin type, or both [38, 40]. Data on the use of phototherapy in children with AD are limited, and therefore, caution must be exercised when this technique is used in children.

### Systemic antihistamines

Scratching of the skin triggers the release of histamine and other mediators, which intensifies itching and can lead to frustrating conditions for patients, including disruptions in sleep. To address this issue, both sedating and non-sedating oral antihistamines are commonly recommended. However, non-sedating antihistamines have not been found to be very effective in managing pruritus (itching), whereas sedating antihistamines such as hydroxyzine, diphenhydramine, and doxepin have shown some benefits in helping AD patients sleep better [41]. It is important to note that there is currently a lack of evidence to fully support the usefulness of antihistamines specifically for AD patients. Antihistamines are an important therapeutic class of drugs in children. Since pediatric population encompasses a wide age group, drug therapy in children should be evidence-based. As many novel antihistamines have now been introduced with additional properties and improved safety profile, it is imperative to raise concerns precluding the routine use of first-generation antihistamines in children. Further research with newer antihistamines focusing on specific pediatric age groups is warranted.

### Roles of multidisciplinary health care providers in management of AD

#### Psychological and behavioral support

Psychologists play a vital role in providing support to patients with AD and their families. They assist in identifying the behavioral and emotional triggers that contribute to itching and scratching, helping patients understand and break this cycle. Psychological interventions have shown promise, although controlled trials in this area are limited.

These interventions include relaxation training, diversion techniques, addressing habit problems (identifying situations that worsen symptoms like scratching and replacing them with healthier responses), and stress management [42–46]. For children with AD, methods such as diversion and redirection to practical activities (such as squeezing a stress ball, engaging in coloring or painting, or applying moisturizer) can be helpful [47, 48]. Various children-friendly apps for relaxation therapy and sleep are also available to aid to improve AD treatment. Psychological involvement also improves the children’s attitude to adopt various approaches to decrease anxiety vis-à-vis stinging with baths and the use of creams [47–50]. Psychologists can be of great help to patients suffering from AD as they help build in them confidence and an acceptance to the changes which may occur due to AD. It is important to note that the effectiveness of these psychological interventions may vary from person to person, and further research is needed to fully establish their impact on AD management.

### Nutritional evaluation and management

In the multidisciplinary therapy of infants and children with AD, a licensed dietician plays a vital role, particularly when the AD patient has concurrent food allergies, which occurs in approximately 35% of newborns and children with moderate-to-severe eczema symptoms [51]. Long-term avoidance of multiple dietary allergens can have adverse effects, including growth failure, below-average height, weight reduction, inadequate nutrient intake, and nutritional deficiencies [52–58]. In addition, small stature has been linked to insufficient sleep-in children with AD. Dietary counseling has been demonstrated to help children with food allergies improve their overall energy consumption, weight, length/height, and micronutrient intake [59].

## Nanotechnological approaches: for effective treatment of atopic dermatitis

**Fig. 3:**
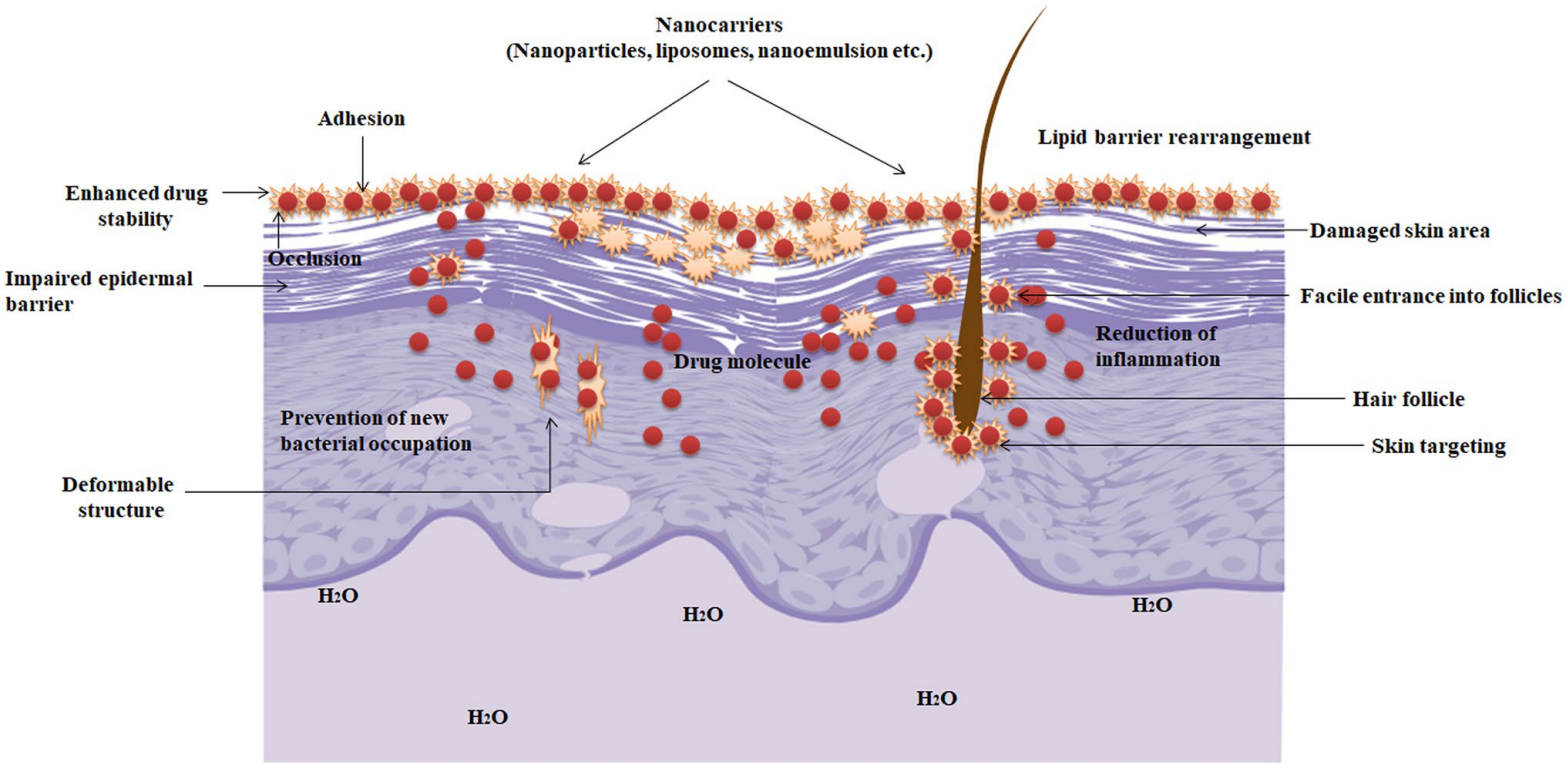
Mechanism of action of the nanocarriers and their interaction with AD skin

Nanotechnology presents a safer and more proficient drug approach for several dermatological conditions such as AD, psoriasis, eczema, and cancer [60, 61]. Nano-cosmetics though are commercialized and available to the end user; however, their therapeutic capability for skin ailments still needs to be explored [62]. Targeted distribution to the skin is made possible by nano-based drug delivery systems using a regulated release profile and diffusion. Due to its site-specific targeting, additional reductions in off-target side effects have also been accomplished. In addition, the use of nanoparticles as a potential drug delivery strategy to circumvent skin’s poor permeability and poor drug solubility has been suggested [62]. Various nanoparticulate formulations have been intended for drug delivery via the topical route for AD, e.g., antibiotics, corticosteroids, herbal, synthetic, and a combination of herbal-synthetic drugs [63]. A diagrammatic representation of nanoparticle types used in drug delivery via topical route is shown in Fig. 3.

Nanoparticles, such as solid lipid nanoparticles (SLNs), nanolipid carriers (NLCs), polymeric nanoparticles (PNPs), nanogels, nanoemulsions (NE), and other nanocarriers, have been shown to be effective delivery options for AD. Traditionally, topical delivery systems include ointments, creams, and gels in some form. Different carrier systems have been suggested to improve the penetration of drugs into the skin, facilitating their retention and, in some cases, enabling controlled release mechanisms [12, 63]. Skin permeation is important for a variety of issues, including contamination by bacteria and chemicals, drug release to the skin (dermatological management), skincare, and safety (cosmetics) [62]. The physicochemical characteristics of nanocarrier systems, including nanoparticle composition, structural design, size, shape, surface charge, and any associated molecules on the surface, determine the interaction with biological systems and nanocarrier cell internalization. Below are several nanocarriers that have proved crucial in reducing AD symptoms by delivering therapeutic actives through topical routes (Table 1).

**Table 1:** Topical nanoformulations developed for atopic dermatitis

Sr. no	Encapsulated drug	Nanocarrier system	List of polymer/lipid	Preparation technique	References
Polymeric nanoparticles					
1	Betamethasone	Polymeric nanoparticles	Hyaluronic acid and chitosan	High-pressure homogenization evaporation	[64]
2	Hydrocortisone	Polymeric nanoparticles	Poly(-caprolactone)	Modified solvent displacement	[65]
3	Tacrolimus	Polymeric nanoparticles	Hyaluronic acid and chitosan	High-pressure homogenization evaporation method	[66]
4	Tacrolimus and nicotinamide	Polymeric nanoparticles	Chitosan	Ionic gelation method	[67]
5	Hydrocortisone and Hydroxytyrosol	Polymeric nanoparticles	Chitosan	Ionic gelation method	[68–70]
6	Hydrocortisone	Polymeric nanoparticles	Chitosan	Ionic gelation method	[71–73]
7	Ceramide and C-phycocyanin	Polymeric nanoparticles	Poly lactic-co-glycolic acid (PLGA) and polyvinyl alcohol	Oil-in-water emulsification method	[74]
8	Budesonide	Polymeric nanoparticles	Chitosan, PLGA and poloxamer	Emulsification/solvent evaporation process	[75]
9	Minerals (calcium, magnesium, sodium, potassium, zinc, and strontium)	Polymeric nanoparticles	Poly (maleic anhydride-alt-butyl vinyl ether) 5% grafted with monomethoxy poly(ethyleneglycol) 2000 MW (PEG) and 95% grafted with 2-methoxyethanol (VAM41-PEG)	Mini-emulsion/solvent evaporation process	[76]
10	Human neutrophil elastase inhibitor (ER143)	Starch-based nanoparticulate system	Caprylic/capric triglyceride and starch	Emulsion-solvent evaporation method	[77]
11	Betamethasone valerate	Polymeric nanoparticles	Chitosan	Nanoencapsulation	[78]
12	Eugenol	Nanocapsules	Anionic methacrylate polymer Eudragit^®^ S100	Encapsulation	[79]
13	Cyclosporine A	Nanocapsules	PLGA	Encapsulation	[80]
14	Meloxicam	Nanocapsules	poly-e-caprolactone, sorbitan monostearate and Miglyol 810^®^	Encapsulation	[81]
Lipid nanoparticles					
15	Cyclosporin A	Solid lipid nanoparticles	Tricaprin and Distearoyl-phosphatidylethanolamine-N-poly(ethylene glycol) 2000 (DSPE-PEG)	Hot homogenization	[82]
16	Tacrolimus	Solid lipid nanoparticles	Stearic acid and poloxamer 188	Modified emulsification and low-temperature solidification	[83]
17	Tacrolimus	Solid lipid nanoparticles	Glyceryl trimyristate (Dynasan 114)	High-pressure homogenization	[84–86]
18	Tacrolimus	Modified nanolipid carrier	Glyceryl trimyristate (Dynasan 114)	High-pressure homogenization	[87, 88]
19	Curcumin	Solid lipid nanoparticles	Precirol ATO 5	Probe ultrasonication method	[89]
20	Tetrahydrocurcumin	Solid lipid nanoparticles	Compritol^®^ 888 ATO	Micro-emulsification technique	[90, 91]
21	Ebastine	Solid lipid nanoparticles	Compritol^®^ 888 ATO	High-speed homogenization	[92]
22	Betamethsone dipropionate	Nanostructured lipid carriers	Glycerylmonostearate (GMS), oleic acid (OLA), and cremophor RH 40	Encapsulation	[93]
23	Halobetasol propionate	Nanostructured lipid carriers	Glyceryl distearate and capric glyceride	High-pressure homogenization	[94]
24	Hydroxyzine HCL	Solid lipid nanoparticles	Glyceryl dibehenate	Double emulsification	[95]
Liposomes and nanovesicles					
25	Tacrolimus	Ethosomes	Lipoid S and ethanol	Mechanical dispersion and ultrasonication method	[101]
26	Betamethasone valerate/diflucortolone valerate	Polymeric liposomes	Lipoid S100, phospholipon 90 G and cholesterol	Thin-film hydration method	[102]
27	Peptide (AT1002) and anti-nuclear factor-kappa B	Liposome	1,2-Dioleoyl-sn-glycero-3-phosphoethanolamine (DOPE) and cholesteryl hemisuccinate (CHEMS)	Small uni-lamellar vesicle (SUV) fusion method	[103–106]
28	Taxifolin glycoside	Liposome	Phosphatidylcholine, Tween 80, *N*-[4-(*p*-maleimidophenyl)butyryl]-phosphatidylethanolamine (MPB-PE), and Pep-1 peptide	Reverse-phase evaporation method	[107]
29	Oregonin	Liposome	Soybean phosphatidylcholine and Tween 80	Reverse-phase evaporation method	[108]
30	Hirsutenone	Liposome	Phosphatidylcholine and Tween 80	Thin-film hydration procedure	[109]
31	Polyvinyl-pyrrolidone	Liposome	Phosphatidylcholine	Polyvinyl-pyrrolidone (PVP)-iodine preparation method	[110]
32	Glycyrrhizin	Liposome	PEG-7 glyceryl cocoate	Film method with high-pressure homogenizer	[111]
33	Cobalamin/vitamin B12	Liposome	l-α-Phosphatidylcholine (soy hydrogenated, HSPC) and cholesterol	Thin-film hydration	[112]
34	Cetirizine/levocetirizine dihydrochloride	Liposome	Phospholipon 90G and Span 80, stearylamine	Thin-film hydration method	[113, 114]
35	IL-13 anti-sense oligonucleotide	Liposome	1,2-Dioleoyl-3-trimethylammonium propane chloride (DOTAP) and sodium cholate	Extrusion method	[115]
36	Adipose stem cell-derived protein extract	Pro-liposomes	Soy phosphatidyl choline and ethanol	Evaporation on matrix	[116]
37	Astaxanthin	Liposomal formulation	Phosphatidylcholine and ethanol	High-pressure homogenization	[117]
38	Piperine	Ethosomes	Phosphatidylcholine and ethanol	Cols method and probe sonicator	[118]
39	Cyclosporin A	Lipid vesicles	Ethanol and phospholipids	Rotary evaporator	[119]
40	Vitamin B12	Lipid vesicles	Phospholipone 90G and cholesterol	Film-hydration method	[120]
Nanoemulsions					
41	Ceramide-3B and ceramide-3	o/w nanoemulsion	Cholesterol, and palmitic acid	High-pressure homogenization	[121]
42	Dimeric ceramides	Microemulsion	Hydrolite 5, isopropylpalmitate, ethanol and sucrose laurate L 595	Micro-emulsification	[122]
43	Rice bran oil	Nano-emulsion	Surfactants sorbitan oleate/PEG-30 castor oil,	Emulsion Phase Inversion (EPI) method	[123]
44	Prednicarbate	Nano-emulsion	Phytosphingosine, Lipoid E80, eutanol	High-pressure homogenization	[124, 125]
45	Oat-derived phytoceramides, lecithin-based microemulsions, and starch-based nanoparticles	Nano-carriers	1, 4-Dioxane, Pluronic^®^ F-127 and 1,2-pentanediol	Emulsification solvent evaporation method	[126]
46	Pioglitazone	Nano-emulsion	Castor oil, labrasol^®^ as surfactant, transcutol^®^ P and propylene glycol	Emulsification	[127, 128]
47	Linseed oil	Nano-emulsion	Pluronic^®^ F-68), Transcutol^®^ HP and Labrafil^®^ M 1944 CS	Ultrasonic emulsification	[129]
48	Nicotinamide	Nano-emulsion	DHA oil	Emulsification	[130]
Miscellaneous system					
49	Tacrolimus	Mesoporous silica nanoparticles	Cetyltrimethylammonium bromide (CTAB), Poloxamer 407 (F-127), Tetraethyl orthosilicate (TEOS)	Modified Stöber method	[131]
50	7,3′,4′-Trihydroxyiso-flavone	Nanosuspension	Polyvinyl-pyrrolidone K30	Solvent-free method (Planetary ball mill)	[132]

### Polymeric nanoparticles

Polymeric nanoparticles offer a versatile approach for delivering therapeutic compounds in dermatological applications. These nanoparticles can either encapsulate the therapeutic agents within their polymeric core or have them adsorbed onto their surface. Nanocapsules and nanospheres are two distinct morphological structures commonly used. The use of polymeric nanoparticles has demonstrated significant potential for topical drug delivery. Pandey et al. developed chitosan nanoparticles coated with hyaluronic acid and loaded with betamethasone valerate (BMV-HA-CNPs). These nanoparticles exhibited enhanced drug penetration and showed promising efficacy in treating AD [64]. Similarly, Rosado et al. employed a modified solvent displacement process to fabricate hydrocortisone-loaded poly (-caprolactone) nanoparticles (PCL-NPs). These nanoparticles demonstrated prolonged drug release and reduced side effects, offering a valuable approach to managing AD [65].

Zhuo and colleagues formulated tacrolimus-loaded chitosan nanoparticles decorated with hyaluronic acid (HA) and demonstrated that HA coating improved dermal targeting and resulted in superior anti-dermatitis efficacy [66]. Yu et al. conducted a study to evaluate the feasibility of chitosan-based nanoparticles loaded with a combination of tacrolimus and nicotinamide (FK506-NIC-CS-NPs). The objective was to investigate the potential of these nanoparticles in reducing or preventing adverse reactions associated with high doses of tacrolimus, especially during long-term therapy [67]. The researchers employed a BALB/c mouse model of AD induced by 1-chloro-2,4-dinitrobenzene (DNCB) and compared the effects of FK506-NIC-CS-NPs to a commercially available ointment (Protopic^®^). The results showed that FK506-NIC-CS-NPs increased skin permeation by 92.2%, facilitating enhanced delivery of FK506 into the deeper layers of the skin. This suggests the potential of these nanoparticles as a promising approach for improving the efficacy of tacrolimus treatment in AD.

Siddique and colleagues conducted a study where they developed nanoparticles to assess the ability of targeted delivery to inflamed skin [68–70]. In a mouse model, they applied cationic polymeric chitosan-based nanoparticles (CSNPs) loaded with hydrocortisone and hydroxytyrosol (HC-HT-CSNPs) topically. These nanoparticles demonstrated excellent penetration into the epidermal and dermal layers, reaching deeper regions of the skin with 2.46 times higher efficiency compared to the commercially available product. No toxic effects were observed in the dermal tissue.

Hussain et al. investigated the pharmacological effects of chitosan nanoparticles loaded with hydrocortisone (HC-CNPs) in a murine model of AD induced by 2,4-dinitrofluorobenzene (DNFB) [71–73]. In both serum and skin samples, the formulation effectively inhibited inflammatory processes, reducing the release of IgE, IL-4, IL-5, IL-6, IL-13, IL-12p70, IFN-γ, TNF-α, histamine production, prostaglandin-E2 expression, and vascular endothelial growth factor (VEGF). Histological analysis also revealed suppression of fibroblast infiltration and elastic fiber fragmentation.

Shin and colleagues developed chitosan nanoparticles that were skin-sensitive and delivered ceramide and C-phycocyanin to provide anti-inflammatory effects without causing cytotoxicity. They evaluated the impact of these nanoparticles on SC in a rat model of AD [74]. The study found that the effect of poly(lactic-co-glycolic acid) nanoparticles containing ceramide on SC formation, as determined by the production of keratinization factors, was similar to or higher than that of free ceramide.

In a different study, Campos et al. [75] encapsulated the anti-inflammatory drug budesonide (BUD) in chitosan (CS)-coated PLGA nanoparticles, which were incorporated into poloxamer hydrogels to enhance anti-inflammatory efficacy and reduce side effects [75]. The nanoparticles were prepared using the emulsification solvent evaporation method and showed no adverse effects on primary human fibroblasts and keratinocytes. However, all formulations generated reactive oxygen species. Although the nanoparticles were unable to penetrate the stratum corneum of excised human skin, nanoencapsulation improved BUD absorption, making it a promising approach for delivering glucocorticoids to the skin of AD patients [75].

After loading with PNPs, Dessy and colleagues developed nanoparticles loaded with anti-inflammatory Dead Sea Water (DSW) minerals, including calcium, magnesium, sodium, potassium, zinc, and strontium. These nanoparticles were prepared using a combined mini-emulsion/solvent evaporation technique, where Poly(maleic anhydride-alt-butyl vinyl ether) grafted with monomethoxy poly(ethylene glycol) 2000 MW (PEG) and 2-methoxy ethanol (VAM41-PEG) were used. The release of DSW minerals from the nanoparticles exhibited a progressive pattern, reaching a plateau phase after approximately 30 h, indicating the diffusion of minerals across the oil–water interface [76].

Marto and colleagues developed a promising strategy for topical delivery of a synthetic human neutrophil elastase inhibitor (ER143) in conjunction with a starch-based nanoparticulate system (StNC), which resulted in improved skin permeability and/or retention. Their formulation underwent skin permeability and retention tests, demonstrating positive outcomes [77].

Shadab and colleagues achieved nanoencapsulation of betamethasone valerate into chitosan-based nanoparticles (BMV-CS-NPs) for enhanced dermal targeting and improved skin permeation. The formulation exhibited satisfactory results, including optimum entrapment efficiency and loading capacity [78].

De Araújo Lopes and co-workers investigated the antioxidant and anti-inflammatory properties of eugenol for the treatment of AD. They evaluated the nanocapsulation of eugenol using an anionic methacrylate polymer called Eudragit^®^ S100. The nanocapsules successfully prevented cytotoxicity in keratinocytes, reduced ear thickness in mice, and decreased MPO activity, as well as IL-6 and KC (CXCL 1) concentrations, suggesting the potential of eugenol as a bioactive molecule for improved skin permeation and irritation prevention [79].

Badihi et al. developed a topical formulation of Cyclosporine A (CsA) nanocapsules (NCs) for the management of severe and persistent AD. The in vitro tests demonstrated the biological activity of encapsulated CsA on mouse splenocytes, including cell proliferation inhibition and suppression of interleukin (IL)-2. Ex vivo experiments on human skin organ culture showed a significant reduction in pro-inflammatory cytokine production. The CsA-NCs topical formulation exhibited increased efficacy in terms of improving skin barrier integrity, reducing systemic pro-inflammatory indicators, and alleviating skin inflammation [80].

Mroginski Weber and colleagues investigated the effect of meloxicam-loaded nanocapsules (M-NCs) using poly-e-caprolactone in an AD mouse model induced by DNCB. They assessed inflammatory factors such as edema and myeloperoxidase (MPO) activity, as well as oxidative factors including thiobarbituric acid reactive species (TBARS) and non-protein thiol (NPSH) levels. The M-NCs formulation effectively reversed skin severity scores, scratching behavior, and inflammatory reactions induced by DNCB, indicating the potential of meloxicam-loaded nanocapsules to alleviate inflammation and improve symptoms in AD [81].

### Lipid nanoparticles

Due to their widespread acceptance as safe, biocompatible, and scale-up-friendly lipid nanocarriers, a wide range of therapeutic agents, from biotechnological products to tiny pharmacological molecules, are increasingly being transported and delivered using these carriers. NLCs incorporate small amounts of liquid lipids into their structure to produce a rearrangement of the lipid matrix preventing drug expulsion and increasing drug loading capacity and long-term stability of the nanoparticles. Solid lipid nanoparticles SLNs consist of lipids solid at room temperature with crystal lipid matrices.

Kim and colleagues prepared SLNs loaded with cyclosporin A using a hot homogenization method. This formulation showed improved skin dispersion, resulting in a twofold increase, and decreased release of inflammatory markers such as IL-4 and IL-5 by TH2 cells when tested in a mouse model of AD [82]. In another study, Kang and co-authors demonstrated enhanced drug transport of thermosensitive SLNs loaded with tacrolimus (TCR-SLNs) compared to a commercial formulation (0.1% Protopic®) in a murine model. TCR-SLNs allowed for deeper penetration of tacrolimus into the skin layers [83].

Pople and Singh [84] utilized the high-pressure homogenization method to prepare tacrolimus lipid nanoparticles (T-LN) using trimyristin as a solid lipid. In vivo dermatopharmacokinetics studies in guinea pigs showed a 3.02-fold higher skin bioavailability compared to the commercially available ointment (Protopic^®^). Gamma scintigraphy in albino rats demonstrated 1.5-fold faster penetration of radioactivity into the skin for T-LN, with limited localization in the target skin area and no widespread distribution to other body organs, indicating its potential for targeted delivery [84–86]. Furthermore, this group enhanced the drug loading of tacrolimus using a modified nanolipid carrier and demonstrated increased drug deposition in albino rats, with significantly elevated drug levels in all skin layers compared to the reference Protopic® ointment. The study also revealed efficient suppression of inflammatory responses in a mouse model of AD [87, 88].

Shrotriya and co-workers prepared the SLNs of curcumin by employing the probe ultrasonication method for the treatment of contact dermatitis. After incorporation into the gel system, it showed enhanced skin deposition, efficient occlusion properties, improvement in antioxidant properties, and inhibition of tyrosinase enzyme action in comparison to conventional curcumin-plain gel [89]. Kakkar and co-authors prepared the tetrahydrocurcumin (THC) loaded lipidic nanoparticles by microemulsification technique [90]. It was found that THC-SLNs gel showed a significantly higher drug release and better (*p* ≤ 0.001) activity in comparison to free THC. As inflammation is intrinsic to all skin ailments, the developed product discovers new therapeutic pathways for numerous skin disorders. They also displayed the enhanced bioactivity of THC-SLNs in *Lacca* mice model of AD and established the complete healing of inflamed skin [91].

Kazim and colleagues developed SLNs loaded with ebastine (E-SLNs) and incorporated them into a hydrogel using chitosan as a gelling agent and glutaraldehyde as a crosslinker. The in vitro drug release studies conducted on the E-SLNs dispersion and E-SLNs loaded hydrogel demonstrated sustained release, with maximum release percentages of 82.9% and 73.7%, respectively, at the end of 24 h. In a mouse model of allergic contact dermatitis (ACD), the topical application of E-SLNs loaded hydrogel alleviated ACD symptoms, as evidenced by reduced swelling, mast cell count, and histological improvements in the ear tissue [92].

Pa and Mm used heat homogenization and sonication to produce betamethasone dipropionate-loaded NLCs with oleic acid (OLA). OLA has been demonstrated to help drugs penetrate deeper into the skin layers. When examined with ex vivo skin penetration testing, betamethasone dipropionate synthesized in NLCs was found to penetrate skin layers more efficiently than conventional cream [93]. NLCs using a higher concentration of OLA had better skin penetration and more medication passed through the skin layers [93]. Similarly, Carvajal-Vidal et al. used a high-pressure homogenization approach to make a halobetasol propionate (HB)-loaded lipid nanocarrier (HB-NLC). The HB-NLC showed an anti-inflammatory impact since it reduced the production of interleukins in keratinocytes and monocytes, indicated by in vitro and in vivo experiments. HB-NLC was found to be an alternative treatment to treat skin inflammation [94].

El-Telbany and colleagues formulated a transdermal gel containing Hydroxyzine HCL (HHCL)-SLNs to enhance the permeation of HHCL through SC and evaluate its peripheral H1-antihistaminic activity against allergic skin conditions. The anti-pruritic efficacy of the formulation was demonstrated using a 2,4-Dinitrochlorobenzene-induced AD lesion model in mice, and the results were further supported by histopathological examinations. Furthermore, the study investigated the effect of HHCL transdermal gel loaded with SLNs on the levels of IL-4 and substance P. The findings revealed a decrease in the levels of IL-4 and substance P, indicating the potential of the formulation in modulating the inflammatory response associated with AD [95].

### Liposomes, nanoliposomes, and nanovesicles

Due to skin barrier destruction, AD patients with stratum spinosum and stratum granulosum appear to have an abundance of lamellar, ovoid, membrane-coating granules (MCGs), which are made up of extruded and parallel discs that aid in the creation of continuous lamellae [96–100]. Liposomes can be used to treat AD because of their comparable structure, which has a moisturizing impact on the SC as well as the ability to transport bioactive chemicals [100]. In the early 1990s, the first unique study on liposomal research was published. Elias [101] developed tacrolimus-loaded ethosomes for dermal delivery and assessed their particle size and entrapment efficiency. The study observed that ethosomes exhibited significantly higher entrapment of tacrolimus, enabling its permeation through the stratum corneum to reach the target site in AD [101].

Eroglu and colleagues formulated betamethasone valerate/diflucortolone valerate-loaded chitosan-based liposomes, demonstrating the safety and efficacy of the formulation in a mouse model of AD [102]. In addition, Ibaraki and colleagues investigated the positive outcomes of topical therapy using small interfering RNA (siRNA) combined with AT1002 peptide, a skin-permeable anti-nuclear factor-kappa B (NF-κB) (RelA)-encapsulated 1,2-dioleoyl-sn-glycero-3-phosphoethanolamine/cholesteryl hemisuccinate (siRelA-DOPE/CHEMS) liposome. The AT1002 peptide was selected for its ability to open and modulate tight junctions. The product underwent in vitro and in vivo testing (including animal and human studies) and was found to be safe and effective [103, 104].

Another peptide option for AD treatment is Pep-1. Kang and colleagues investigated the production of a peptide-conjugated elastic liposomal preparation (composed of phosphatidylcholine, polysorbate 80, *N*-[4-(*p*-maleimidophenyl)butyryl]-phosphatidylethanolamine or MPB-PE) of taxifolin glycoside (TXG-Pep1-EL) in an NC/Nga mice model. The study demonstrated optimal skin delivery, permeation, and retention, as well as improvements in skin functions such as hydration, elasticity, and immune responses [107].

Kang et al. developed elastic and highly flexible liposomes loaded with oregonin (ORG) using soybean phosphatidylcholine and Tween 80 (85:15 w/w %) (ORG-EL). These liposomes exhibited a fourfold higher deformability index compared to marketed liposomes. To enhance skin permeability, the peptide Trans-activating transcriptional activator (Tat), known for its ability to open tight junctions, was incorporated (ORG-EL-Tat). The study demonstrated remarkable skin penetration of the nanocarriers, and the combination with Tat peptide further enhanced it. Moreover, significant reductions in the levels of inducible nitric oxide synthase (iNOS), cyclooxygenase-2 (COX-2), IL-4, IgE, and eosinophils in the skin and blood were observed [108].

In another study by Kang and colleagues, liposomes composed of phosphatidylcholine and Tween 80 (85:15 w/w %) (HST-EL-Tat) were developed to facilitate the skin delivery and penetration of hirsutenone, a natural immune modulator. This ad hoc developed liposomal formulation led to a reduction in Inducible nitric oxide synthase (iNOS), cyclooxygenase-2 (COX-2), IL-4, IL-13, IgE, and eosinophils in a murine model, indicating its efficacy in modulating the immune response [109].

Augustin et al. investigated the anti-septic and anti-inflammatory efficacy of a liposomal polyvinylpyrrolidone (PVP)-iodine hydrogel (3%) applied topically for 4 weeks in a prospective, single-arm (uncontrolled), open-label phase II pilot clinical trial involving 20 AD patients. The treatment resulted in significant improvements in “Global Clinical Severity” (GCS) scores, pain levels, quality of life, and “Eczema Area and Severity Index” (EASI) scores. The product exhibited mild adverse reactions and was well tolerated [110].

Another potential agent for AD treatment is glycyrrhizin, an antioxidant and antimicrobial compound extracted from licorice. Glycyrrhizin, specifically its active component 18β-glycyrrhetinic acid (GA), was loaded into liposomes and examined for its efficacy. In vivo, research using a mouse model confirmed the effectiveness of GA in preventing itching in chronic dermatitis [111].

Jung et al. investigated the topical delivery of cobalamin/vitamin B12 by loading it into a liposomal hydrogel of adenosylcobalamin (Lipo-AdCbl) using the thin film hydration method. This formulation significantly enhanced skin permeability up to 17 times and effectively alleviated symptoms and immune responses in an NC/Nga mice model [112].

Goindi and colleagues developed liposomes by combining cetirizine/levocetirizine dihydrochloride, a second-generation antihistamine, with Phospholipon®90G and edge activators. In a mouse model, these liposomes exhibited superior penetration capabilities and led to a significant reduction in cutaneous eosinophil count, erythema, and irritation compared to commercially available ointments and creams [113, 114].

Kim et al. focused on IL-13 anti-sense oligonucleotide and its complexation with cationic elastic liposomes (IL-13-ASO-cEL). The formulation was found to effectively suppress IL-13 production and release by up to 70%, in addition to IL-4 and IL-5, demonstrating its potential for modulating immune responses [115].

Jahn et al. developed a liposomal formulation capable of serving as a carrier for stem cells or pure stem cell extracts. They created pro-liposomes using soy phosphatidylcholine, poloxamer-407, ethanol, and sorbitol (AAPE) after loading them with protein extracts derived from advanced adipose stem cells. These pro-liposomes exhibited excellent stability and favorable physical–chemical properties [116].

Lee et al. developed a liposomal formulation of Astaxanthin (L-AST) for the treatment of AD and evaluated its effects on a phthalic anhydride (PA)-induced AD in a male SKH-1 mice model. In the PA-induced skin condition, L-AST demonstrated a reduction in AD-related inflammatory mediators and markers induced by iNOS and COX-2. L-AST therapy also restored oxidative stress balance and enhanced the production of antioxidant proteins, such as glutathione peroxidase-1 (GPx-1) and heme oxygenase-1 (HO-1), in skin tissues. Moreover, L-AST treatment resulted in decreased STAT3 and NF-B transcriptional activity in PA-induced skin tissues, suggesting its potential benefits over free AST for AD treatment [117].

Kumar et al. investigated the efficacy of a cream containing ethosomes of piperine for the treatment of AD. The ethosome-based cream exhibited enhanced deposition in the epidermis and dermis. In an in vivo efficacy study using a BALB/c mice model, the ethosomal cream showed significant reductions in ear and skin thickness, skin severity, and levels of white blood cells, granulocytes, and IgE antibodies [118].

Verma and Fahr developed a lipid combination (NAT-8539) to improve the topical distribution of cyclosporin A (NAT-8539-CsA) by incorporating it into vesicles ranging from 56.6 to 100.6 nm in size. Vesicles containing CsA and 10 or 20% ethanol exhibited enhanced deposition of CsA into the SC compared to vesicles prepared without ethanol [119].

Guillot et al. formulated novel lipid vesicles for encapsulating vitamin B12 and improving its skin penetration in AD. These lipid vesicle formulations demonstrated effective delivery of cyanocobalamin into the deeper layers of the skin. The authors suggested that this approach holds promise for the topical administration of vitamin B12 as an excellent nitric oxide scavenger in AD treatment [120].

### Nanoemulsions (NE) and microemulsions

Nanoemulsions are emulsions characterized by the presence of nanosized oil globules dispersed in an aqueous phase. The oily phase can be formulated using various lipids and oils, such as triglycerides and essential oils, resulting in nanoemulsions with diverse physicochemical and biological properties. The aqueous portion of the nanoemulsion can also be modified by incorporating different water-soluble components. One key distinction between nanoemulsions and microemulsions is that the former requires energy input during production, while the latter forms spontaneously.

Yilmaz and Borchert conducted a study on positively charged o/w nanoemulsions (PN) containing ceramide-3B, ceramide-3, cholesterol, and palmitic acid (PNSC). The efficacy of PNSC cream was compared to PN, negatively charged o/w nanoemulsion (NNSC), and stratum corneum lipids stabilized with Carbopol-940 (carbomer) and a marketed cream (Physiogel^®^). Fourteen healthy female participants aged 25–50 were involved in the testing. All formulations exhibited improvements in skin moisture and elasticity, with PNSC demonstrating significantly higher efficacy than PN and NNSC. This outcome highlighted the importance of phytosphingosine, lipids, and ceramides in the formulation [121]. Similarly, Neubert et al. developed three types of nanoformulations containing dimeric ceramides: a colloidal microemulsion, a ceramide-based cream with ethoxy diglycol as a penetration enhancer, and a nanoemulsion. While the colloidal microemulsion displayed reduced penetration and permeation characteristics, it significantly improved the physicochemical characterization of the drug [122].

Bernardi et al. investigated the effectiveness of rice bran oil nanoemulsions, prepared using a low-energy spontaneous emulsification technique. These nanoemulsions, comprising 10% rice bran oil, 10% sorbitan oleate/PEG-30 castor oil, 0.05% antioxidant, and 0.50% preservatives in distilled water, exhibited low irritation, good stability, enhanced skin hydration, and maintained the pH of the skin within the standard range [123]. Baspinar et al. utilized high-pressure homogenization to formulate positively charged prednicarbate nanoemulsions. The resulting formulation was deemed theoretically suitable and stable for patients with AD. The increased penetration of the nanoemulsion was attributed to the enhanced interaction between the positively charged nanoemulsion and the negatively charged corneocytes of the SC, which serves as the primary barrier of the skin [124, 125].

In a study conducted by Tessema et al., they utilized Carbopol®980 as a gelling system to create nanocarriers loaded with oat-derived phytoceramides (CER). They also developed microemulsions of lecithin and starch-based nanoparticles as drug delivery systems. The researchers demonstrated the effectiveness of their formulations in repairing the skin barrier. They observed that the lecithin-based microemulsion gel exhibited enhanced permeation of oat CERs into the deeper layers of the skin and improved in vitro penetration compared to the starch-based nanoparticle gel [126].

In another investigation by Espinoza et al., they focused on the delivery of pioglitazone NE, a peroxisome proliferator-activated receptor agonist (PGZ-NE), for the treatment of AD. The researchers reported a significant reduction in IL-6, IL-1, and TNF-α levels following the application of PGZ-NE [127]. Furthermore, PGZ-NE exhibited stability, hyperbolic kinetics, and high retention potential. The in vivo efficacy was confirmed through a decrease in lesions, enhancement of skin barrier function, reduced infiltration of inflammatory cells, and decreased production of pro-inflammatory cytokines in an oxazolone-induced mouse model [128].

Kıldacı et al. developed and analyzed nanoemulsion (NE) formulations containing Linum usitatissimum seed (linseed) oil (LSO) to explore their potential for treating AD in vitro and in silico. The researchers employed the ultrasonic emulsification method to produce LSO-NE. The mutagenicity of LSO-NE was evaluated using an in vitro Ames/Salmonella assay, which indicated no mutagenic effects on Salmonella typhimurium strains TA98 and TA100. LSO was identified as a promising therapeutic candidate for the treatment of AD [129].

In a study by Pradeep and Viswanad, a DHA oil-based nicotinamide nanoemulsion gel was formulated for the treatment of AD. In vitro drug release was higher compared to a marketed formulation. Anti-inflammatory cell line studies demonstrated the nanoemulsion gel's ability to inhibit COX activity, LOX activity, inducible nitric oxide synthase, and cellular nitrate levels [130].

### Miscellaneous systems

In a study conducted by Parekh et al. mesoporous silica nanoparticles (TMSNs) loaded with tacrolimus were developed to address the challenges associated with its solubility and effective topical distribution. These TMSNs were then incorporated into a Carbopol gel and subjected to various gel characterization tests. The in vivo tests were conducted on Balb/c mice with dermatitis induced by 1-fluoro-2,4-dinitrobenzene (DNFB). Both in vitro and in vivo studies demonstrated that the TMSNs-loaded gel exhibited significantly higher retention of tacrolimus (ex vivo rat skin) and led to a significant reduction in ear thickness and improved histology in the mice (in vivo) [131]. In another study by Huang et al., nanosized particles of 7,3′,4′-trihydroxyisoflavone (734THIN) were produced using a solvent-free technique and a planetary ball mill. Polyvinyl-pyrrolidone K30 was employed as an excipient. The resulting nanosuspension effectively inhibited the "Mitogen-Activated Protein Kinase" (MAPK)-related signaling cascade, leading to reduced expression of COX-2 and metalloproteinase (MMP)-9 [132].

## Clinical trials for topical treatment of AD

To date, clinical trials are focused on finding more efficacious and safer therapies to combat the inflammation and itch of AD. In addition, they provide an assessment of the duration of the skin lesions and clinical remission obtained after treatment. The clinical advancement of topical treatments for AD has been initiated based on findings that topically applied drugs can penetrate the deepest layers of the skin. Table 2 provides an overview of the clinical studies conducted on different drugs used in the treatment of AD [133].

**Table 2:** Clinical status of topical treatment of atopic dermatitis

Therapeutic agent/product developed	Objective	Outcomes	Clinical status/verified date
Corticosteroids/calcineurin inhibitors/chemical salts			
10% imidazole-4-carboxylic acid cream	To illustrate the efficacy, safety and tolerability of imidazole-4-carboxylic acid	Topical atopic dermatitis severity index (toADSI) score and visual analogue scale for pruritis (VAS) score was observed	Phase 2 (completed)/February 2010
Alefacept	Assessed the efficacy, safety, and tolerability of alefacept	The safety profile of alefacept was observed with 75% or greater improvement in Psoriasis Area and Severity Index (PASI) at any time	Phase 4 (completed)/March 2011
Elidel (pimecrolimus cream, 1%) hylatopic plus emollient foam (non-medicated device)	Comparing the efficacy of Hylatopic™ Plus Emollient Foam™ and Elidel® (pimecrolimus) cream 1%	Improved and maintained the signs of eczema (erythema, population/infiltration, lichenification, and scaling/dryness) with twice daily Elidel applied topically to one side of the body as compared to three times days Hylatopic Plus applied topically to the other side of the body	Phase 4 (completed)/April 2011
AMG 157	Assessed the tolerability, safety, and pharmacokinetics of AMG 157	Eczema Area and Severity Index (EASI) score, investigator’s global assessment (IGA) score and serum PK parameters were observed	Phase 4 (completed)/May 2011
DNK333 (5 mg)	Evaluated the efficacy and safety of DNK333	Efficacy of DNK333 in pruritus reduction in atopic dermatitis patients measured by actigraphy and visual analogue scale (VAS)	Phase 2 (completed)/July 2012
Pimecrolimus	Explored the safety of pimecrolimus cream 1% in the long-term treatment of AD in comparison to topical corticosteroids	For adults with moderate AD, pimecrolimus cream 1% is well tolerated, reduces the incidence of flares, reduces/eliminates corticosteroid use, improves long-term disease control, and enhances the patient’s quality of life	Phase 4 (completed)/December 2012
OTC Cream, Cosmetic Eczema Cream, 0.05% Desonide Cream	Explored the tolerability and efficacy of new topical formulations in treating AD	Desonide cream/lotion 0.05% (DesowenTM) is an effective and safe modality of treatment in Indian patients suffering from mild to moderate steroid-responsive dermatoses	Phase 4/April 2014
Hydrocortisone 2.5% ointment or triamcinolone acetonide 0.1% ointment	Described the efficacy of two corticosteroid-based ointment	Hydrocortisone 2.5% ointment and triamcinolone 0.1% ointment achieved effective skin concentrations greater than the effective concentration achieved by oral prednisone	Study completed/July 2014
Topical amino acid cream and desonide	Comparative influences of an amino acid moisturizing cream and desonide cream	Change in erythema, pruritus, exudation, excoriation, and Lichenification from baseline of atopic dermatitis target lesions observed after application of either amino acid moisturizing cream or desonide cream twice daily for 3 weeks	Phase 3/November 2014
BPR277 ointment (controlled application)	To study the safety and tolerability of the ointment	Systemic and local tolerability of BPR277 ointment, as measured by a change in local tolerability score, number of adverse events, and clinically significant changes in standard hematology, and blood chemistry	Phase 1/February 2015
CT327 ointment	Assessed the safety, efficacy, and tolerability of the ointment	CT327 showed effectiveness in reducing pruritus in subjects with atopic dermatitis using a pruritus visual analogue scale (VAS)	Phase 2/November 2015
Fluocinonide cream	To depict the tolerability and efficacy of fluocinonide cream 0.1% (Vanos®)	Investigator’s global assessment of atopic dermatitis integrates all lesions for the overall score. This is used to measure the disease severity and most resemble assessments performed in the clinic setting i.e., the score ranges from ‘0’ = clear to ‘5’ = very severe disease	Recruiting participants/December 2015
QGE031, cyclosporine A	To emphasize the efficacy and safety of QGE031	Change in eczema area and severity index (EASI) was observed and efficacy responses were assessed using EASI	Phase 2/April 2016
AQX-1125	To estimate the effect of 12 weeks of treatment with once-daily administration of AQX-1125 compared to placebo	The evaluation of the treatment effect of once-daily administrations of AQX-1125 compared to placebo over 12 weeks on the COPD Assessment Tool (CAT) score was observed	Phase 2 (completed)/June 2017
Baricitinib	Assessed the efficacy and safety of baricitinib in combination with topical corticosteroids (TCS)	The primary endpoint was the proportion of patients achieving a validated investigator global assessment for atopic dermatitis (vIGA-AD) score of 0 (clear) or 1 (almost clear), with a 2-point or greater improvement from baseline at week 16	Phase 3 (completed)/September 2019
Baricitinib	Investigated the efficacy and safety of baricitinib as monotherapy in participants with moderate-to-severe atopic dermatitis	Baricitinib was efficacious for patients with moderate-to-severe AD with no new safety findings over 16 weeks	Phase 3 (completed)/January 2020
Phosphodiesterase 4 (PDE4) inhibitors/anti-inflammatory agent/phototherapy			
Apremilast	To express the efficacy of apremilast in patients	Percentage change from baseline was observed in the eczema area and severity index (EASI) score at week 12 and score ranges were from 0 to 72	Phase 2 (completed)/November 2010
GW842470X cream	To explore the clinical effectiveness and tolerability of 3% (w/w) GW842470X cream	Clinical efficacy of 3% GW842470X cream applied to the skin of adult patients with moderate atopic dermatitis using the Eczema Area Severity Index (EASI)	Phase 2 (completed)/March 2012
0.5% Roflumilast cream	Evaluated the effect of topical roflumilast reducing the lesions of AD	Modified local SCORAD is the sum of 5 individual indexes; erythema, edema/ papulation, oozing/crusts, excoriations, and lichenification scored on a 4-point scale, where 0 = absent and 3 = severe, with a total possible score of 15. Higher scores indicate greater severity	Phase 4 (completed), June 2015
GSK2894512 1% Cream, GSK2894512 0.5% Cream	To examine the safety and efficacy of two concentrations (0.5% [%] and 1%) and two application frequencies (once a day and twice a day)	The percentage of participants who have an investigator global assessment (IGA) score of clear or almost clear (0 or 1) at week 12 and a minimum 2-grade improvement in IGA Score from baseline to week 12 for intent to treat (ITT) population was obtained	Phase 2/February 2016
Photocil	Assessed the safety and efficacy of Photocil	Percent repigmentation and increased patient compliance	Phase 4 (completed)/February 2016
Crisaborole 2% topical ointment [EUCRISA]	Evaluated the safety and efficacy of crisaborole ointment, 2%	Total sign score (TSS) assesses the severity of stasis dermatitis lesions. TSS = sum of scores from all clinical signs; ranging from 0 (none) to 12 (most severe), where a higher score indicated greater severity	Recruiting participants/July 2019
Biological therapy/antibiotics/JAK inhibitors/GPCR19 agonist			
Ustekinumab	To calculate the skin thickness reversal and the immune mechanism during the treatment with Ustekinumab	Greater improvement was observed from their baseline objective SCORAD (SCORing Atopic Dermatitis) at Week 16	Phase 2/April 2016
HAT01H Cream	Testing the efficacy and safety of topical HAT01H in patients with moderate-to-severe AD	The primary efficacy was determined by absolute change from baseline to week 12 in inflammatory SCORAD	Phase 1 (completed)/November 2018
Secukinumab	Investigated the effects of a new treatment called secukinumab in patients with AD	The proportion of patients with a reduction of the eczema score EASI of at least 50% was observed	Phase 2 (completed)/May 2019
Tradipitant	Multi-center, randomized, double-blind, placebo-controlled study to be conducted in the United States in subjects with AD	Reduction of worst itch in atopic dermatitis was observed as measured by numerical rating scale (NRS)	Phase 3 (completed)/April 2020
PF-04965842	Evaluated the efficacy and safety of PF-04965842 in adolescent participants 12 to < 18 years of age with moderate-to-severe AD	The percentage of participants achieving eczema area and severity index (EASI) response of > = 75 percent (%) improvement from baseline at week 12 was revealed	Phase 3 (completed)/June 2020
Upadacitinib	Assessed the efficacy and safety of upadacitinib for the treatment of adolescent and adult participants with moderate-to-severe AD	Safety and efficacy, including 75% improvement in the eczema area and severity index and validated investigator global assessment for atopic dermatitis score of clear (0) or almost clear (1) with 2 or greater grades of improvement, were assessed	Phase 3 (completed)/June 2020
Lebrikizumab	Evaluated the safety and efficacy of lebrikizumab compared with placebo in patients with moderate-to-severe atopic dermatitis	The percentage of participants achieving eczema area and severity index (EASI-75) (≥ 75% reduction from baseline in EASI score) at week 16 was observed	Phase 2 (completed)/July 2020
Upadacitinib	Measured the safety and efficacy of multiple doses of upadacitinib monotherapy versus placebo in the treatment of adults with moderate-to-severe atopic dermatitis (AD)	Upadacitinib monotherapy showed improvement in skin clearance and reduction in itch compared to placebo at week 16	Phase 2 (completed)/July 2020
BI 655130, Spesolimab	Determined the safety, tolerability, and efficacy of BI 655130 in patients with AD	Percentage change from baseline in the eczema area and severity index (EASI) score at week 16 was observed	Phase 2 (completed)/July 2020
PF-06700841	Explored the efficacy, safety, tolerability, and PK of multiple topical formulation concentrations of PF-06700841 topical cream	The severity of clinical signs of AD (erythema, induration/papulation, excoriation, and lichenification) scored separately for each of 4 body regions(head and neck, upper limbs, trunk [including axillae and groin] and lower limbs [including buttocks]) on 4-point scale: 0 = absent; 1 = mild; 2 = moderate; 3 = severe	Phase 2 (completed)/July 2020
GPCR19) agonist HY209	Efficacy of HY209 Gel in Healthy Male Volunteers for Atopic Dermatitis	Incidence of treatment-emergent adverse events [upto Day 8 (single dosing), upto Day 38 (multiple dosing)] and number of participants with abnormal laboratory values and/or adverse events that are related to treatment was obtained	Phase 1 (completed)/January 2021
PF-04965842	JAK1 inhibitor with medicated topical therapy in adolescents with Atopic Dermatitis (JADE TEEN)	The EASI quantifies the severity of AD based on both severity of lesion clinical signs and the percent of body surface area (BSA) affected. The EASI score can vary in increments of 0.1 and range from 0.0 to 72.0, with higher scores representing greater severity of AD	Phase 3 (completed)/June 2021
Herbal products			
Colloidal oatmeal (Topical cream)	Established the efficacy and safety of an over-the-counter cream in children having mild to moderate AD	Colloidal oatmeal is well tolerated by AD patients, as it has a very low risk of skin irritation and treatment-emergent adverse effects	Phase 4 (completed)/June 2012
Vitamin D_3_	To assess the effects of vitamin D supplementation on several key aspects of the immune system of children with AD	Vitamin D deficiency and insufficiency in children with allergic diseases can be treated with maximal recommended doses of vitamin D3 for a short period of time, especially if they were prescribed with inhalation or intranasal corticosteroids	Not applicable (completed)/February 2015
Vitamin D_3_	To study the effect of oral Vitamin D_3_ on the antimicrobial peptide expression in the skin or saliva of subjects	Relative abundance of cathelicidin (CAMP) messenger ribonucleic acid (mRNA) in lesional and non-lesional skin for atopic dermatitis participants who received oral vitamin D3 versus vitamin D3-Placebo	Phase 2/June 2015
Indigo naturalis ointment	To evaluate the safety and efficacy of indigo naturalis ointment	Indigo naturalis ointment is effective for treating mild to severe AD topically and appears to be safe	Phase 2 (completed)/February 2017
Melatonin	To establish whether supplementing melatonin is effective in improving sleep problems in children with AD	Objective sleep measures as measured by actigraphs and SCORAD Actigraph parameters including sleep latency, sleep efficiency, total sleep time, and wake episodes and duration; SCORAD and objective SCORAD for disease severity assessment was also done	Phase 2 (completed)/April 2015
SAN007 (5%) cream	Tolerability and efficacy trial of SAN007 (5% East Indian sandalwood oil in a cream formulation) treatment regimen	Safety was assessed by evaluating adverse events (AEs) with respect to severity, duration, and relationship to the study drug	Phase 2 (completed)/May 2019
Role of behavior			
Behavioral: text messages	To determine if sending text messages with treatment reminders and facts regarding AD to caretakers of children with AD will have a positive effect on the disease severity	Patient’s EASI Score (0–72) was determined at the initial and follow-up exam. Mean differences in EASI scores between arms will be assessed using t-tests since EASI scores are used to measure the severity of a patient’s atopic dermatitis	Not applicable (completed)/August 2017

## Patents

Patents are proof of novelty and innovation leading to the development of new formulations. A range of drugs including phytosterols, anti-microbials, anti-inflammatory medicines, corticosteroids, and antihistamines have in reported in different patents related to AD. Various types of nanocarriers have been utilized to deliver these drugs in the context of AD. These nanocarriers include nanoemulsions, PLGA nanoparticles, biodegradable polymeric carriers, SLNs, surface-modified multi-layered nanostructures, and photoactive plasmonic nanoparticles. These nanocarriers have shown great promise in the field of drug delivery for the treatment of AD [133]. Several patents dating from 2010 to 2021 related to the field have been collated in Table 3 [134], suggesting the commercial possibility of nanocarriers via topical application. These patents have underlined the preparation techniques with the development of nanoparticle production and their applicability to AD disease [134].

**Table 3:** Patents on the topical administration of nanocarriers in the treatment of atopic dermatitis

Patent number, year	Title of the patent	Inference
WO 2010051918 A2, 2010	Compositions depicting lipid-based nano/microparticles for enhanced dermal action	Defined a dermal composition useful in skin irritation, or to treat skin disorders based on synergistic action exerted by metallic particles
EP 2310000 A1, 2011	Dermal delivery	Highlighted the methods and systems to treat skin disorders involving the use of nanoemulsions
US 20110135742 A1, 2011	Controlled release loaded anti-inflammatory and anti-bacterial nanoparticles	Invention revealed the development of nanoparticles acquiring antimicrobial and anti-inflammatory activity
US 20110236491 A1, 2011	Topical anti-inflammatory composition	Depicted the methods and compositions to treat inflammation
US 8003127 B2, 2011	Nanoparticulate corticosteroid and antihistamine formulations methods of making, and methods of administering thereof	Described compositions contacting nanoparticulate carriers for delivering corticosteroid and antihistamines
EP 2419535 A1, 2012	Delivery of oligonucleotide-functionalized nanoparticles	Related to methods and compositions to deliver oligonucleotide-functionalized nanoparticle
EP 2583671 A1, 2013	Colloidal nanoscale carriers for active hydrophilic substances and method for producing the same	Described a polymeric colloidal nanocarriers and its development process for the controlled delivery of hydrophilic agents
EP 2667844 A2, 2013	Nanoparticles based on dermal and systemic delivery of drugs	Invented a cosmetic composition containing poly (lactic glycolic) acid (PLGA) NPs for topical delivery. Further, it also specified polymeric NPs for topical application
US 20130202712 A1, 2013	Compositions and methods for treating or preventing immuno-inflammatory disease	Described the methods and compositions for treating immuno-inflammatory conditions consisting of a polyphenolic phytoalexin compartmentalized in a biocompatible and/or biodegradable polymeric carrier
US 20130236571 A1, 2013	Dispersions in oil of dead sea nanosized material preparation and uses thereof	Disclosed a formulation containing a dispersion of dead sea material in solid nanoparticles. Further, also depicted methods of treating and/or preventing skin diseases topically
EP 2667859 A2, 2013	Nanoparticle compositions, formulations thereof, and uses thereof	Depicted composition for the preparation of nanoparticles and systems and methods for utilizing them in the treatment of disorders
US 8647661 B1, 2014	Surface-modified multi-layered nanostructures for dermal delivery	Depicted the development of surface-modified multi-layered nanostructures for topical delivery
WO 2014043304 A1, 2014	Topical compositions and methods of use	Comprised of methods and compositions containing fruit extracts obtained from *Synsepalum dulcificum* tree, showed in vitro anti-inflammatory, antimicrobial, and spermicidal activity
WO 2014145749 A1, 2014	Targeted polymeric inflammation-resolving NPs	Depicted NPs contain a target element that binds to cells, tissues, or organs, selectively, a diagnostic agent, an outside “stealth” layer, and a biodegradable polymeric material. These NPs were developed to provide targeted delivery in inflammation
EP 2688560 A2, 2014	A composition containing lipid nanoparticles	Invented a pharmaceutical formulation containing a therapeutically active ingredient incorporated as a solid solution or dispersion in lipid nanoparticles
US 8715736 B2, 2014	Nanoparticle formulations for skin delivery	Methods and formulations of nanostructured lipid carrier for treating skin conditions
US 9198853 B2, 2015	Methods and systems for treating inflammatory diseases using nitric oxide	Described the compositions and methods for treating inflammatory diseases
EP 2838509 A1, 2015	Formulation development and method for treating inflammatory skin diseases	Topically used compositions containing corticosteroids and methods for treating inflammatory skin disease
EP 2874663 A1, 2015	Nanoconstructs with pharmacological activity	The invention related to methods for development and active substances delivery in the cell cytosol. Further, it depicted the development of nanoconstructs for the treatment of inflammatory diseases
CN 105188687 A (2015)	METADICHOL R liquid and gel nanoparticle formulations	Provided methods for regulating physiological and metabolic parameters and for the treatment of diseases using metadichol as a liquid or gel formulation
WO 2015031189 A1, 2015	Targeted delivery of nanoparticles to the skin surface	Described improved transport of compositions containing photoactive plasmonic nanoparticles and light for the treatment of skin diseases
WO 2015072846 A1, 2015	Chitosan nanoparticle for skin-targeted drug delivery system and its method	Disclosed is a chitosan-based carrier prepared with antioxidants and anti-inflammatory agents for treating atopic dermatitis
WO 2015140722 A1, 2015	Aptamers for topical delivery	Methods for topical application of aptamers were described
WO 2016011049 A2, 2016	Compositions and methods for disease treatment using nanoparticle-delivered compounds	Depicted the methods and compositions for the treatment of skin diseases using NPs
US20170266292 A1, 2017	Lipidic compound-telodendrimer hybrid nanoparticles	Explained the lipid hybrid nanoparticles for skin inflammatory disease
CA2946982 A1, 2018	Nystatin nanosuspension formulation using high-pressure homogenization	Depicted the method of formulation for skin infection
KR101923969 B1, 2018	Nanovesicles derived from Propionibacterium bacteria	Depicted the nanoparticles derived from a bacterium belonging to the genus Propionibacterium, and treating the atopic dermatitis
CN105663027 A, 2018	External preparation containing sirolimus as well as a preparation method	Described novel preparation capable of permeating skin includes solid lipid nanoparticles, sirolimus solid lipid nanoparticles gel
BR102018072899 A2, 2020	Nanostructured pharmaceutical composition containing dilapiol for topical administration in the treatment of skin infections	Demonstrated the topical administration in the treatment of skin infections
CN108283621 B, 2020	Nasal cavity nano-preparation mometasone furoate liquid crystal gel nanoparticle and preparation method	Showed the effects of resisting inflammation, resisting allergy
US20200188429 A1, 2020	Novel gold-based nanocrystals for medical treatments and electrochemical manufacturing processes	Described the use of gold nanocrystals or suspensions or colloids thereof for the treatment or prevention of skin inflammatory (including chronic inflammatory) conditions, autoimmune conditions, hypersensitivity reactions, and/or cancerous diseases or conditions
JP6700297 B2, 2020	A composition for preventing or treating inflammatory diseases, which comprises extracellular vesicles derived from lactic acid bacteria as an active ingredient	Used for preventing, ameliorating, or treating an inflammatory disease containing extracellular vesicles derived from lactic acid bacteria as an active ingredient, and a method for diagnosing atopic dermatitis
US10537640 B2, 2020	Ultrasound delivery of nanoparticles	Enhanced delivery of compositions for treatment of skin tissue with photoactive plasmonic nanoparticles
AU2021107029A4, 2021	Method for the treatment of immunological disease using nanoparticles	Enhanced immunity using nanoparticles in immunological disease
CN113712828A, 2021	Pickering emulsion with a relieving effect via transdermal absorption	Nano-scale Pickering emulsion is beneficial to the transdermal absorption of active ingredients and improved the bioavailability
CN108186472B	Anti-allergy relieving composition, anti-allergy relieving emulsion, and preparation method of anti-allergy relieving emulsion	Reduced the allergy and adverse reactions caused by stimulation and shows the synergistic enhancement effects

## Safety facets and issues related to nanotechnological approaches

As there is a remarkable use of nano-tailored drug delivery, the main concern is their safety. Interaction of nanoparticles with cells induces immunological responses at different levels. Negatively charged nanoparticles have superior tolerability in comparison to their positively charged particles [135]. Nanoparticles with a negative charge and a mean diameter smaller than 200 nm have demonstrated a significant reduction in the production of pro-inflammatory mediators when administered simultaneously with DNFB, a Th1-cell sensitizer [136]. This effect is attributed to the inhibition of mast cell degranulation and interactions with other immune cell types. Additional research has investigated the immune responses triggered by the application of free DNFB. It has been observed that keratinocytes exhibit increased production of cytokines (such as IL-1β and IL-18), which are responsible for promoting mast cell activation and degranulation, leading to histamine release [135–137].

The toxicity of nanoparticles can be evaluated at various levels, including molecular, cellular, tissue, and organ levels [138]. This toxicity depends on factors such as nanoparticle loading capacity and whether they are in a free form. Smaller nanoparticles have demonstrated a greater ability to penetrate the skin, allowing for increased systemic circulation and potential interaction with other organs and tissues. However, their small size can also lead to harmful effects in vivo due to enhanced cellular interactions. Protein aggregation is commonly observed in the vicinity of nanoparticle surfaces [139].

Nanoparticles can induce cell toxicity through the generation of ROS via Fenton’s reaction. ROS production is detrimental and can result in the disruption of lysosomal membranes, leading to the release of enzymatic hydrolytic machinery, iron cations, protons, and other factors. This, in turn, can lead to mitochondrial dysfunction, protein aggregation, and increased cellular oxidative stress [140, 141]. Therefore, toxicological studies play a crucial role in assessing the potential toxicity of nanoparticles [139]. It is essential that nanomedicines comply with current safety regulations to mitigate any potential risks of toxicity. Moreover, modifying the physicochemical properties of nanoparticles, particularly their size, is a significant aspect of the development of nanoparticles without compromising their safety [142, 143].

## Regulatory potential and future remarks

Despite the growing interest in developing nanoparticulate formulations to enhance the permeation and bioavailability of drugs administered through the skin, there are currently no specific guidelines issued by the FDA and European Medicines Agency (EMA) for topical application. The existing regulatory framework for nanomedicine products primarily focuses on parenteral formulations. However, aspects of this framework can be considered when identifying and investigating the critical quality attributes of nanoformulations in general, including their physical, chemical, and microbiological characteristics during pharmaceutical development [144–146]. For topically applied products, additional specifications can be obtained from the FDA’s draft guidance on nanomaterials contained in drug products [147, 148].

Due to the increased interest in biomedical applications of nanotechnology products and the well-established differences in the biopharmaceutical performance between nanoformulations and bulk materials, regulatory agencies in the US and Europe have urged manufacturers to conduct more comprehensive pre-authorization studies to assess the quality, safety, and efficacy of new nanomedicine products. When the surface of a nanomaterial is coated with a ligand, the nanoformulation becomes more complex, requiring additional information. EMA has issued a reflection paper on the surface coating that provides guidelines for the full characterization of coating materials and their impact on the performance of nanoformulations [149]. The extent of the required studies varies depending on the type and size of the ligand, such as whether it is a small molecule, peptide, protein, or antibody. More complex ligands necessitate more detailed studies.

The regulatory framework for topically applied nanomedicine is currently not well established and is primarily associated with fewer requests for marketing authorization. Therefore, further studies are needed to demonstrate the mechanisms of penetration and define the quality profile of such products in order to facilitate their development. Moreover, due to the higher costs associated with their development and manufacturing, the rationale for a nanomedicine product heavily relies on demonstrating its clinical superiority over existing therapeutic alternatives available on the market.

## Conclusion

Numerous factors, consisting of epidermal gene mutations, dysfunction of the skin barrier, immune dysregulation, inflammation of nervous tissues, the altered composition of lipids, and microbial imbalance, are factors involved in the development of AD. Several approaches have been employed to repair the skin barrier function and manage skin inflammation in patients with AD. To surmount the drawbacks of topically applied anti-inflammatory agents and systemic immunosuppressants, an extensive attempt has been devoted to establishing new therapeutic choices i.e., biologics and microbiome transplantation. Besides, AD development may be prevented using moisturizers and probiotics with a high probability in infants. Further progress in our perception of AD pathophysiology will permit us to attain an accurate medicine advance in the treatment of AD.

With the assistance of resourceful nanocarriers, novel approaches can be demonstrated by combining with new administration routes for the successful optimization potential of skin-targeted nanoparticulate systems for AD management. Nanotechnological application in skin ailments has offered a promising and potential response to resolve the issues with skin inflammatory diseases. To revolutionize the aspects of clinical dermatology, novel nanomedicine-based techniques have been predicted. Nanomedicines as drug carriers offer superior activity including enhancement in therapeutic efficacy with minor toxicity by small dose, drug localization, and drug-specific targeting. Nevertheless, most existing studies lack clinical data on AD thus the need for research directed toward the clinical examination to explore the outcome of nanoparticles as future anti-AD nanocoutured therapy. Ensuring the compliance of nanoformulations with current safety regulations is crucial to mitigate potential risks of toxicity. It is important to emphasize the modification of the physicochemical properties of nanoparticles, with size being a particularly significant aspect, during the development of nanoparticles. This modification should be done in a way that does not compromise the safety of the nanoparticles. Furthermore, in vitro*/*in vivo correlations for topically applied nanosystems and regulatory guidelines to assess their technological and biopharmaceutical properties would aid in clinical translation and marketing authorizations of these products in the future. The nanotechnological-based drug delivery system would ultimately become a significant accomplishment to the treatments accessible to AD patients in the near future.

Furthermore, in vitro*/*in vivo correlations for topically applied nanosystems and regulatory guidelines to assess their technological and biopharmaceutical properties would aid in clinical translation and marketing authorizations of these products in the future. The nanotechnological-based drug delivery system would ultimately become a significant accomplishment to the treatments accessible to AD patients in the near future.

## Data Availability

This article has no additional data.

## Abbreviations

AAD: American Academy of Dermatology
ACD: Allergic contact dermatitis
AD: Atopic dermatitis
AFU: Adult fingertip units
AST: Astaxanthin
ASO: Anti-sense oligonucleotide
BMV: Betamethasone valerate
BSA: Body surface area
CAT: COPD assessment tool
CHEMS: Cholesteryl hemisuccinate
COX-2: Cyclooxygenase-2
CR: Ceramide
CsA: Cyclosporin A
CS: Chitosan
CTAB: Cetyltrimethylammonium bromide
DNCB: 1-Chloro-2,4-dinitrobenzene
DOPE: 1,2-Dioleoyl-sn-glycero-3-phosphoethanolamine
DSPE: Distearoyl-phosphatidylethanolamine
DSW: Dead sea water
FLG: Filaggrin
GCS: Global clinical severity
GMS: Glycerylmonostearate
GPx-1: Glutathione peroxidase-1
HB: Halobetasol propionate
HC: Hydrocortisone
HHCL: Hydroxyzine HCL
HO-1: Heme oxygenase-1
iNOS: Inducible nitric oxide synthase
INF-γ: Interferon-gamma
IGA: Investigator’s global assessment
IL-1: Interleukin-1
IL-1β: Interleukin-1β
IL-4: Interleukin-4
IL-5: Interleukin-5
IL-6: Interleukin-6
IL-8: Interleukin-8
IL-10: Interleukin-10
IL-12p70: Interleukin-12p70
IL-13: Interleukin-13
IL-18: Interleukin-18
IL-22: Interleukin-22
ITT: Intent to treat
KLK: Kallikrein
LCs: Langerhans cells
MAPK: Mitogen-activated protein kinase
MCGs: Membrane-coating granules
MDLC: Langerhans cells (LCs) monocyte-derived LC-like cells
MMP: Matrix metalloproteinase
mRNA: Messenger ribonucleic acid
MPO: Myeloperoxidase
NLCs: Nano-lipid carriers
NCs: Nanocapsules
NE: Neutrophil elastase
NF-κB: Nuclear factor-kappa B
NIC: Nicotinamide
NPSH: Non-protein thiol
OLA: Oleic acid
ORG: Oregonin
PASI: Psoriasis area and severity index
PCL: Poly(-caprolactone)
PEG: Poly(ethylene glycol)
PGZ: Pioglitazone
PLGA: Poly lactic-co-glycolic acid
PNPs: Polymeric nanoparticles
PVP: Poly vinyl pyrrolidone
ROS: Reactive oxygen species
SC: Stratum corneum
SCORAD: Scoring atopic dermatitis
SLNs: Solid lipid nanoparticles
SUV: Small uni-lamellar vesicle
StNC: Starch-based nanoparticulate system
TCR: Tacrolimus
TBARS: Thiobarbituric acid reactive species
TMSNs: Mesoporous silica nanoparticles loaded with tacrolimus
THC: Tetrahydrocurcumin
TEOS: Tetraethyl orthosilicate
TEWL: Transepidermal water loss
Th: T helper
TNF-α: Tumor necrosis factor-alpha
TSLP: Thymic stromal lymphopoietin
TSS: Total sign score
UVA: Ultraviolet A
UVB: Ultraviolet B
USFDA: United State Food and Drug administration
VAS: Visual analogue scale
VEGF: Vascular endothelial growth factor
toADSI: Topical atopic dermatitis severity index
vIGA-AD: Validated investigator global assessment for atopic dermatitis
